# Mechanism of Anemic Murmurs

**Published:** 1890-12

**Authors:** DeLancey Rochester

**Affiliations:** Buffalo, N. Y.; Lecturer on Physical Diagnosis, Medical Department University of Buffalo; Physician to Department of Diseases of the Chest, Fitch Dispensary; 469 Franklin Street


### MECHANISM OF ANEMIC MURMURS.[1]

By DeLancey Rochester, M. D., Buffalo, N. Y.

Lecturer on Physical Diagnosis, Medical Department University of Buffalo; Physician to Department of Diseases of the Chest, Fitch Dispensary.

When fluid is driven with a certain amount of velocity through a constricted opening into a larger space beyond, a sound is produced which has been shown by careful experiments to depend upon the vibrations of what have been termed fluid veins, produced at a

1. Read before the Medical Club.

greater or less distance from the orifice, according to its size and the velocity with which the fluid is propelled. In the case of heart murmurs dependent upon organic lesions — whether the murmur in question is direct or regurgitant — the sound is produced in the same way, the blood being propelled with a certain velocity through a constricted opening into a larger space beyond. The vibrations of these fluid veins are transmitted to the surrounding blood, to the walls of the containing vessel, and thence through whatever medium may intervene, to the ear of the listener.

This is, beyond doubt, the true explanation of the mechanism of heart murmurs dependent upon valvular disease. But there are other conditions in which heart murmurs occur, in which there is evidence from both ante-mortem and post-mortem observation that no valvular lesion exists.

It is to the murmurs, cardiac, arterial, and venous, which accompany anemia in its various forms, to which I invite your attention this evening. Let us first consider the cardiac murmurs and decide, if possible, how they are produced, and then push our investigation to the murmurs as heard over the arteries and veins. Before answering the question as to *how* the anemic heart murmurs are produced, it behooves us to observe *where* they are produced, as this will aid us greatly in answering the first question. With the exception of Laennic, Guttman, and, in one case, Strümpell, all observers whose works I have been able to consult, agree that the anemic heart murmur is systolic in rhythm, and the majority of these observers agree that it is basic in situation. Walshe says:

> This murmur is, as far as I have observed, invariably basic in seat and systolic in time, produced at the orifices of the aorta and of the pulmonary artery — with a force at each proportional to the power of its communicating ventricle; scarcely conducted along the aorta at all; frequently audible, on the contrary, at the second left or pulmonary cartilage; only in exceptional cases audible below the nipple; and never, within my experience, perceptible as far as the left apex.

Roberts says of the anemic cardiac murmur:

> This has usually the position of a pulmonary systolic murmur. . . It may, however, be situated over the aorta or be heard at all the orifices.

Flint says, speaking of the diagnosis of anemia:

> A frequent sign is a systolic bellows murmur, referable to the carotid arteries. Not less frequently, systolic murmurs are heard at the base of the heart, produced within the aorta and the pulmonary artery.

Strümpell says of the functional cardiac murmurs:

They are usually heard loudest over the base of the heart in the neighborhood of the pulmonary valves, although sometimes at the apex of the heart. As a rule they are purely systolic in time, but we certainly heard, in one case of pernicious anemia, a loud diastolic murmur of anemic origin.

Thus I might go on quoting one observer after another to show that the anemic murmur has been located at each of the valves of the heart, by far the largest number, placing the point of greatest intensity over the pulmonary or aortic orifice. In my own experience this murmur has had its point of greatest intensity over the aortic valve, and has been transmitted into the subclavian and carotid arteries.

Dr. George William Balfour, of Edinburgh, agrees with other observers that the anemic heart murmur is systolic in rhythm and basic in seat, but places its point of greatest intensity not over the aortic or pulmonary orifice, "but," I quote from his book, "actually about one inch and a half, or rather more, to the *left* of the pulmonary area, and in the same plane immediately over the part where the appendix of the left auricle pops up from behind, just to the left of the pulmonary artery. This so-called arterial murmur is, therefore, not arterial at all, but strictly auricular in its source. This is a most important fact: it at once links this chlorotic murmur with others whose source we distinctly know, and gives us an unmistakable clue to its true point of origin." It is a well-known fact that sometimes a mitral regurgitant murmur is heard with greatest distinctness at the above-mentioned point, the sound being conducted better "along the course of the regurgitating blood, the fluid veins producing sonorous vibrations louder at the point of impingement than at that of origin." From these observations he concludes that the heart murmur of anemia is one of mitral regurgitation.

Having thus seen at how many different points this murmur has been heard, is there any rational manner in which we can explain its production, which will satisfy all these observations, for we obviously have no right to doubt the diagnostic power of any observer unless we have actual proof of the inaccuracy of his observations.

First, then, how is this murmur produced at the aortic and pulmonary orifices? In a healthy individual, by the ventricular systole a certain amount of blood of a definite weight is driven with definite velocity against the semilunar valves, pressing them flat against the walls of the artery. Evidently, if the amount of blood or the weight of the blood is reduced below a certain limit, though the velocity remain the same, the valves will not be driven firmly back against the arterial wall, but will hang out into the lumen of the vessel and thus produce a constricted opening with a larger space — the cavity of the artery — beyond, all that is needed, with a certain velocity of the blood, to produce the fluid veins of Savant and the consequent sonorous vibrations resulting in the murmur. So, whether we have anemia from hemorrhage, in which the actual amount of blood is reduced, or spanemia from any cause, in which the blood is thinned and watery, diminished in weight, we can account for the murmur heard at the pulmonary and aortic orifices by the obstruction of the incompletely opened valves to the current of the blood. But how about the murmur heard at the mitral and tricuspid areas? As they are systolic in rhythm, they must necessarily be regurgitant in character.

In cases of profound spanemia, the whole muscular system becomes greatly debilitated. The heart is the hardest-worked muscle in the body and, along with the rest of the muscular system, becomes greatly weakened, in some advanced cases actually undergoing some fatty degeneration. As a result of this marked debility of the heart we have more or less dilatation of the cavities, due to diminution of elasticity in the muscular fibres of the walls. This produces some dilatation of the auriculo-ventricular orifices, and a consequent insufficiency of the valves, resulting in regurgitation. This is the explanation given by Dr. Balfour of the mitral regurgitation observed by him. That such dilatation, with some hypertrophy, does exist in cases of spanemia, has been proved by experiments upon dogs, and by post-mortem examinations in human beings.

Another method of explaining this regurgitation — one long ago brought forward by Walshe — is through the fatty degeneration and consequent weakening of the musculi-papillares to such an extent as to allow the auriculo-ventricular valves to yield to the pressure of the blood during the ventricular systole and allow the regurgitation to take place.

In those rare cases, such as Strümpell's case of pernicious anemia, where a *diastolic* anemic murmur occurs, the dilatation of the ventricle has probably proceeded so far as to dilate the aortic orifice, and so allow regurgitation to take place at that point.

Thus, we see, we have a rational explanation of the modes of production of anemic heart murmurs, which is applicable to all cases, and is none other than that which holds in the case of organic murmurs, namely, an obstruction to the onflow of the blood, causing at one point a narrowing of the lumen of the vessel through which it passes, and thus producing the fluid veins above referred to. The arterial murmur is synchronous with the systole of the heart, and is, in my opinion, merely a transmission, along the current of the blood, of the cardiac murmur arising at the aortic orifice, such as is almost invariably found in connection with organic obstructive lesion of the aortic valve.

The venous murmur, however, is a *continuous* humming sound, and so cannot be referred for its origin to the cardiac murmur, which is necessarily intermittent, as it occurs only with the systole. This murmur, therefore, must originate in the vein itself, and here we find a cause for it. Like all the other murmurs, it, too, is caused by constriction of the lumen of the containing vessel at one point, leaving a larger space beyond, the obstruction being produced. by the hanging out of the valves, due to the relaxed condition of the walls of the veins. The continuousness of the sound is due to the fact that the velocity of the blood current is much more constant, though not so great, as in the arteries.

Of course, the mode of production of so-called hemic murmers is, and must necessarily always remain, to a certain extent, hypothetical. Still we have the following facts before us: First, that when fluid is forced at a certain velocity through a constricted opening into a larger space beyond, at a certain distance from the orifice its particles are thrown into sonorous vibrations. Second, that in certain diseased states of the cardiac valves, we have, beyond a doubt, the necessary conditions for the production of such sound, and the presence of the sound. Third, that, when there is no such diseased state of the valves, but when, from the impoverished state of the blood, we would expect the healthy valves to be placed in such a position as to produce the before-mentioned constriction, we find the murmur, the indication that such constriction exists, present. Therefore, I think we are perfectly justified in concluding that the valves are in that position, especially as the murmer can be satisfactorily accounted for in no other way.

If this explanation of the mode of production of the cardiac murmur of anemia is true, it enables us to draw certain conclusions of practical value: First, that the so-called functional cardiac murmurs are really organic in origin, though it is not the valve, but the muscle, that is diseased; second, certain organic diseases of the heart are curable, and that, therefore, it behooves us carefully to differentiate our cases, in order to make a true prognosis. Dr. Balfour relates several cases of cardiac murmur in which an unfavorable prognosis had been given by observers of good repute, which, however, he had diagnosed as anemic in origin, and by proper treatment had caused to disappear, the patients regaining complete health and strength.

The chief points in diagnosis of the anemic murmur are its softness and the generally diffused area over which it is heard, together with the presence of the venous hum and the other signs of anemia, including the result of spectroscopic and microscopic examination of the blood.

Of course, it is possible — and not infrequent — for organic heart disease and anemia to co-exist; but we should always bear in mind the fact that the anemia *may* be the prime cause, and consequently we should be very guarded in our prognosis until that condition has been overcome; for, with the overcoming of the anemia, we may find that the heart disease also has been vanquished.

469 Franklin Street.

